# Placental Mitochondrial DNA Content and Particulate Air Pollution during *in Utero* Life

**DOI:** 10.1289/ehp.1104458

**Published:** 2012-05-24

**Authors:** Bram G. Janssen, Elke Munters, Nicky Pieters, Karen Smeets, Bianca Cox, Ann Cuypers, Frans Fierens, Joris Penders, Jaco Vangronsveld, Wilfried Gyselaers, Tim S. Nawrot

**Affiliations:** 1 Centre for Environmental Sciences, Hasselt University, Diepenbeek, Belgium; 2 Belgian Interregional Environment Agency, Brussels, Belgium; 3 Biomedical Research Institute, Hasselt University, Diepenbeek, Belgium; 4 Department of Obstetrics, East-Limburg Hospital, Genk, Belgium; 5 Department of Public Health, Occupational and Environmental Medicine, Leuven University (KULeuven), Leuven, Belgium

**Keywords:** fetal development, mitochondrial DNA content, mitochondrial function, particulate matter

## Abstract

Background: Studies emphasize the importance of particulate matter (PM) in the formation of reactive oxygen species and inflammation. We hypothesized that these processes can influence mitochondrial function of the placenta and fetus.

Objective: We investigated the influence of PM_10_ exposure during pregnancy on the mitochondrial DNA content (mtDNA content) of the placenta and umbilical cord blood.

Methods: DNA was extracted from placental tissue (*n* = 174) and umbilical cord leukocytes (*n* = 176). Relative mtDNA copy numbers (i.e., mtDNA content) were determined by real-time polymerase chain reaction. Multiple regression models were used to link mtDNA content and *in utero* exposure to PM_10_ over various time windows during pregnancy.

Results: In multivariate-adjusted analysis, a 10-µg/m³ increase in PM_10_ exposure during the last month of pregnancy was associated with a 16.1% decrease [95% confidence interval (CI): –25.2, –6.0%, *p* = 0.003] in placental mtDNA content. The corresponding effect size for average PM_10_ exposure during the third trimester was 17.4% (95% CI: –31.8, –0.1%, *p* = 0.05). Furthermore, we found that each doubling in residential distance to major roads was associated with an increase in placental mtDNA content of 4.0% (95% CI: 0.4, 7.8%, *p* = 0.03). No association was found between cord blood mtDNA content and PM_10_ exposure.

Conclusions: Prenatal PM_10_ exposure was associated with placental mitochondrial alterations, which may both reflect and intensify oxidative stress production. The potential health consequences of decreased placental mtDNA content in early life must be further elucidated.

Particulate matter (PM) is a part of ambient air pollution and is most relevant to human health (Brunekreef and Holgate 2002; Nawrot et al. 2011). PM has been associated with adverse health outcomes of the fetus (Ballester et al. 2010; Dejmek et al. 1999; Gemma et al. 2006; Glinianaia et al. 2004; Kannan et al. 2006; Morello-Frosch et al. 2010) and neonate (Scheers et al. 2011). In addition, the functional morphology of the placenta is also influenced by PM exposure in experimental animal models (Veras et al. 2008). The underlying mechanisms by which PM exposure may induce adverse fetal health effects are poorly understood. Several studies have emphasized the importance of PM and its associated metal components in the formation of reactive oxygen species (ROS) (Chahine et al. 2007; Li et al. 1996) and inflammation (Salvi et al. 1999).

The placenta is a metabolically active organ that plays a role in nutrient transfer, growth, and organ development. Mitochondria play an important role in the regulation of these processes. These intracellular organelles are essential for cellular energy provision through the production of adenosine-5´-triphosphate (ATP) via oxidative phosphorylation. Each cell contains approximately 200–2,000 mitochondria, each carrying 2–10 copies of mitochondrial DNA (mtDNA) that are bound to protein structures. The major difference between human nuclear DNA (nDNA) and mtDNA is that the latter lacks protective histones, chromatin structure, and introns. Additionally, the mitochondrial DNA repair mechanisms work less efficiently than that of nDNA (Lee and Wei 2000). Mitochondria are the major intracellular sources and primary targets of ROS, so mtDNA is particularly vulnerable to ROS-induced damage and has a high mutation rate (Linnane et al. 1989). Mitochondria compensate for these mutations, resulting in a change in mtDNA copy number (i.e., change in mtDNA content). Recently, mitochondrial function has been linked to various disease mechanisms and can be assessed by measuring the mtDNA content, an established marker of mitochondrial damage and dysfunction (Hou et al. 2010; Sahin et al. 2011).

Developmental adaptations due to metabolic changes, including suboptimal fetal nutrition, permanently “program” the fetus and may lead to adverse pregnancy outcomes that form the origin of diseases that may arise in adult life (Barker et al. 1991; Geelhoed and Jaddoe 2010; Made et al. 2006). Mitochondrial damage and dysfunction contributes to metabolic shifts and may represent a biological effect along the pathway linking PM to effects on the newborn. However, whether placental and cord blood mtDNA content is associated with PM_10_ (PM with aerodynamic diameter ≤ 10 µm) exposure during *in utero* life has never been studied. In the present study we investigated the association of placental and cord blood mtDNA content with long- and short-term exposure to airborne PM_10_ and residential distance to major roads.

## Material and Methods

*Study population and data collection.* Aging is a complex phenotype responsive to a plethora of environmental exposures from early life onward including particulate air pollution. The current study is part of a new initiated and ongoing birth cohort “ENVIR*ON*AGE” (the acronym emphasizes the environmental influence on the aging process). We recruited 178 newborns (only singletons) from South-East-Limburg Hospital in Genk born between Friday 1200 hours and Monday 0700 hours from 5 February 2010 until 3 April 2011. The only inclusion criterion was that mothers had to be able to fill out questionnaires in Dutch. Enrollment was spread equally over all seasons of the year. The overall participation rate of eligible mothers was 47%. During the first month of the campaign, midwives recorded the reason of nonparticipation. The main reasons (in descending importance) were failure to ask for participation, communication problems, or complications during labor. Participating mothers provided written informed consent when they arrived at the hospital for delivery, and they completed study questionnaires in the postnatal ward after delivery to provide detailed information on age, socioeconomic status, ethnicity, smoking status, place of residence, pregestational body mass index (BMI), and parity. Socioeconomic status was coded and condensed into a scale with scores ranging from 0 to 2 based on mother’s education. Ethnicity was classified based on the native country of the newborn’s grandparents as European (when two or more grandparents were European) or non-European (when at least three grandparents were of non-European origin). Current smokers were defined as having smoked before and during pregnancy. Before-smokers were defined as those who had quit before pregnancy, and never-smokers had never smoked.

Samples of placental tissue (*n =* 174) and umbilical cord blood (*n =* 176) were collected immediately after delivery, along with other perinatal parameters such as newborn’s sex, birth date, birth weight and length, gestational age (range, 35–42 weeks), Apgar score, and ultrasonographic data. All neonates were assessed for congenital anomalies immediately after birth and all were considered healthy. The Apgar score after 1 min ranged from 2 to 10 but improved up to values between 7 and 10 after 5 min for all participants. Birth date was condensed into a seasonal scale where a difference was made between cold periods (October–March) and warm periods (April–September).

The study was conducted according to the principles outlined in the Helsinki Declaration (World Medical Association 2008) for investigation of human subjects. Written informed consent was provided by all study participants in accordance with procedures approved by the Ethical Committee of Hasselt University and South-East-Limburg Hospital.

*Sample collection.* Umbilical cord blood was collected immediately after delivery in Vacutainer^®^ Plus Plastic K2EDTA Tubes (BD, Franklin Lakes, NJ, USA). Blood cell counts (including platelet counts) and differential leukocyte counts were determined using an automated cell counter with flow differential (Cell Dyn 3500; Abbott Diagnostics, Abott Park, IL, USA). Samples were centrifuged at 3,200 rpm for 15 min to retrieve buffy coats and instantly frozen, first at –20°C and afterward at –80°C.

Placentas were obtained for 174 mothers in the delivery room and deep-frozen within 10 min. Afterward, we thawed placentas to take tissue samples for DNA extraction following a standardized protocol as described by Adibi et al. (2009). Briefly, villous tissue, protected by the chorioamniotic membrane, was biopsied from the fetal side of the placenta and preserved at –80°C. We assessed within-placenta variability in a random subset of six placentas by comparing biopsies taken at four standardized sites across the middle region of the placenta, approximately 4 cm away from the umbilical cord. The first biopsy was taken to the right of the main artery and the three other biopsies in the remaining quadrants of the placenta. mtDNA content within each placenta varied by a mean of 19.3% across the quadrants. To minimize the impact of within-placental variability, biopsies used for mtDNA content assays were all taken 1–1.5 cm below the chorioamniotic membrane at a fixed location by using a device to orientate the fetal side of the placenta in relation to the umbilical cord. Care was taken by visual examination and dissection to avoid the chorioamniotic membrane contamination. Each biopsy was approximately 1 to 2 cm^3^. Histological confirmation of cell type in 10 placentas showed consistent results in all studied samples.

*Exposure measurement.* We calculated the regional background levels of PM_10_ for each mother’s home address using a kriging interpolation method (Jacobs et al. 2010; Janssen et al. 2008) that uses land cover data obtained from satellite images. This model provides interpolated PM_10_ values from the Belgian telemetric air quality networks in 4 × 4 km grids. To explore potentially critical exposures during pregnancy, individual PM_10_ concentrations (micrograms per cubic meter) were calculated for various periods: 0–7 days before delivery (lag 0–7), the last month of pregnancy, and for each of the three trimesters of pregnancy, with trimesters being defined as 1–13 weeks (trimester 1), 14–26 weeks (trimester 2) and 27 weeks to delivery (trimester 3). The exposure during the whole pregnancy was also calculated. The date of conception was estimated based on ultrasound data. Additionally, nitrogen dioxide (NO_2_) exposure was interpolated using the same methods as PM_10_ exposure and is used in a sensitivity analysis. Distances from the mother’s residence to a major road were calculated through geocoding (the shortest distance being set at 10 m). A major road was defined as an N-road (major local traffic road with average total number of motor vehicles per 24 hr > 10,000) or an E-road (motorway/highway).

The Royal Meteorological Institute (Brussels, Belgium) provided mean daily temperatures and relative humidity for the study region; these are averaged using the same exposure windows as for PM_10_. The temperature and relatively humidity averaged 10.1 ± 1.4°C and 80.9 ± 10.1%, respectively. Apparent temperature (8.4 ± 1.6°C) was calculated by using the following formula (Kalkstein and Valimont 1986; Steadman 1979):

–2.653 + (0.994 × Ta) + (0.0153 × Td^2^),

where Ta is air temperature and Td is dew-point temperature (degrees Celsius).

*Measurement of mtDNA content.* DNA was extracted from white blood cells of the buffy coat and placental tissue cells using the MagMAX® DNA Multi-Sample kit (Applied Biosystems, Foster City, CA, USA) following the manufacturer’s instructions. Briefly, this purification kit uses MagMAX^TM^ magnetic bead-based nucleic acid isolation technology for producing high quantities of purified DNA. RNA contamination was minimized with an RNase digestion step. The concentration of extracted DNA was measured at 260 nm with the Nanodrop spectrophotometer (ND-1000; Isogen Life Science, De Meern, the Netherlands). Both DNA yield (nanograms per microliter) and purity ratios (A260/280 and A260/230) were determined. Extracted DNA was stored at –20°C until further use.

mtDNA content was measured in placental tissue and leukocytes of umbilical cord blood by determining the ratio of two mitochondrial gene copy numbers [*MTF3212/R3319* (mitochondrial forward primer from nucleotide 3212 and reverse primer from nucleotide 3319) and *MT-ND1* (mitochondrial encoded NADH dehydrogenase 1)] to three single-copy nuclear control genes [*RPLP0* (acidic ribosomal phosphoprotein P0), *ACTB* (beta actin), and *HBB* (hemoglobin beta)] using a quantitative real-time polymerase chain reaction (qPCR) assay. Extracted genomic DNA was diluted to a final concentration of 5 ng/µL in RNase free water, before the qPCR runs. PCR reactions were set up by aliquoting 7.5 µL master mix into each well of a MicroAmp® Fast Optical 96-Well Reaction Plate compatible with the 7900HT Fast Real-Time PCR System (Applied Biosystems), followed by 2.5 µL of each experimental DNA sample, for a final volume of 10 µL per reaction. The master mix consisted of Fast SYBR® Green I dye 2× (Applied Biosystems; 5 µL/reaction), forward (0.3 µL/reaction) and reverse (0.3 µL/reaction) primer and RNase free water (1.9 µL/reaction). Primer sequences (Table 1) were diluted to a final concentration of 300 nM in the master mix. Two nontemplate controls and six inter-run calibrators were carried along in each PCR plate. The thermal cycling profile was the same for all transcripts: 20 sec at 95°C for activation of the AmpliTaq Gold® DNA-polymerase, followed by 40 cycles of 1 sec at 95°C for denaturation and 20 sec at 60°C for annealing/extension. Amplification specificity and absence of primer dimers was confirmed by melting curve analysis at the end of each run (15 sec at 95°C, 15 sec at 60°C, 15 sec at 95°C). After thermal cycling, raw data were collected and processed. C_T_ (cycle threshold)–values of the two mitochondrial genes were normalized relative to the three nuclear reference genes according to the qBase software (Biogazelle, Zwijnaarde, Belgium). The program uses modified software from the classic comparative C_T_ method (ΔΔC_T_) that takes multiple reference genes into account and uses inter-run calibration algorithms to correct for run-to-run differences (Hellemans et al. 2007). Plate effects were minimized by measuring one gene for all 178 placenta or cord blood samples in 1 day. The coefficient of variation for the mtDNA content in inter-run samples was 4.2%.

**Table 1:** Primer sequences for selected genes and their accession number.

Gene	Accession number	Nuclear/ mitochondrial	Forward 5’–3’	Reverse 5’–3’	Primer efficiency (%)
MTF3212/R3319		NC_012920.1		M		CACCCAAGAACAGGGTTTGT		TGGCCATGGGTATGTTGTTAA		96.3
MT-ND1		NC_012920.1		M		ATGGCCAACCTCCTACTCCT		CTACAACGTTGGGGCCTTT		99.3
RPLP0		NM_001002.3		N		GGAATGTGGGCTTTGTGTTC		CCCAATTGTCCCCTTACCTT		100.7
ACTB		NM_001101.3		N		ACTCTTCCAGCCTTCCTTCC		GGCAGGACTTAGCTTCCACA		96.8
HBB		NM_000518.4		N		GTGCACCTGACTCCTGAGGAGA		CCTTGATACCAACCTGCCCAG		100.4
Abbreviations: ACTB, beta actin; HBB, hemoglobin beta; MTF3212/R3319, mitochondrial forward primer from nucleotide 3212 and reverse primer from nucleotide 3319; MT-ND1, mitochondrial encoded NADH dehydrogenase 1; RPLP0, acidic ribosomal phosphoprotein P0. Accession numbers are from the National Center for Biotechnology Information (http://www.ncbi.nlm.nih.gov/).										

*Statistical analysis.* We used SAS software (version 9.2; SAS Institute Inc., Cary, NC, USA) for database management and statistical analysis. Continuous data were checked for normality and are presented as arithmetic means ± SD or geometric means with interquartile range (IQR) when data were not normally distributed. Categorical data are presented as frequencies (percent) and numbers. Pearson or Spearman correlation coefficients and linear regression were used to assess the relationship of mtDNA content from placental tissue or umbilical cord blood with PM_10_ exposure. We performed multiple linear regression to determine the independent variables of mtDNA content. Covariates considered for entry in the model (*p* ≤ 0.10) were newborn’s sex, maternal age, pregestational BMI, net weight gain, socioeconomic status, ethnicity, smoking status, parity, gestational age, season, and time-specific apparent temperature. Newborn’s sex, maternal age, smoking status, gestational age, and ethnicity were forced into the model regardless of the *p*-value. In addition, umbilical cord models were adjusted for white blood cell count, percentage of neutrophils, and platelet counts to account for cord blood cell distribution. Q-Q plots of the residuals were used to test the assumptions of all linear models.

## Results

*Characteristics of the study population.* Table 2 summarizes the characteristics of the 178 mother–newborn pairs. Maternal age averaged 29.1 years and ranged from 18 to 42 years. The mothers had a mean pre-gestational BMI of 24.3 ± 4.8 kg/m^2^. Of the mothers, 15.7% (*n =* 28) smoked during pregnancy, and 29.2% (*n =* 52) had ever smoked. The average pack-years for mothers who ever smoked was 6.1 ± 5.1. Most were working mothers (87.7%), who lived on average 15.5 km (IQR = 5–20 km) from their workplaces. The study population included 82 male and 96 female newborns, and 87.6% (*n =* 156) were classified as Europeans. Seven infants were born preterm (< 37 weeks). Birth weight averaged 3,403 ± 386.7 g. We determined the mtDNA content in cells from placental tissue and cord blood of 174 and 176 subjects respectively.

**Table 2:** Study population characteristics (*n* = 178).

Characteristic	Mean ± SD, geometric mean (25th–75th percentile) or n(%)
Maternal
Age (years)	29.1 ± 4.9
< 20	6 (3.4)
20–29	88 (49.4)
30–35	65 (36.5)
≥ 35	19 (10.7)
Socioeconomic status
Low	28 (15.7)
Middle	58 (32.6)
High	92 (51.7)
Smoking
Never	126 (70.8)
Before pregnancy	24 (13.5)
Before and during pregnancy	28 (15.7)
Cigarettes/day
0	124 (75.6)
1 to 10	28 (17.1)
≥ 11	12 (7.3)
Pack-years of ever smokers	6.1 ± 5.1
Pregestational BMI (kg/m2)	24.3 ± 4.8
Net weight gain (kg)	14.5 ± 6.5
Parity
1	101 (56.8)
2	57 (32.0)
≥ 3	20 (11.2)
Daily apparent temperature (°C)	8.4 ± 1.6
Newborn
Gestational age (weeks)	39.2 (39–40)
Preterm delivery (< 37 weeks)
Yes	7 (3.9)
No	171 (96.1)
Sex
Male	82 (46.1)
Female	96 (53.9)
Ethnicity
European	156 (87.6)
Non-European	22 (12.4)
Season
Cold period	104 (58.4)
Warm period	74 (41.6)
Apgar score
1 min	8.4 (8–9)
5 min	9.5 (9–10)
Neonate birth weight (g)	3,403 ± 386.7
Neonate length (cm)	50 (49–51)
Placental mtDNA content^a^	1.03 (0.6–1.63)
Umbilical cord mtDNA content^b^	1.02 (0.75–1.30)
White blood cells (× 10.e3/μL)	15.3 ± 4.6
Neutrophils (%)	52.3 ± 8.5
Platelets (× 10.e3/μL)	289.4 ± 95.4
Not normally distributed values are presented as geometric means with 25–75th percentile. mtDNA content is determined as mtDNA copy number (mean of MTF3212/R3319 and MT-ND1) normalized to nDNA copy number (mean of RPLP0, ACTB and HBB). aData available for 174 subjects. bData available for 176 subjects.		

PM_10_ exposure averaged 24.9 ± 11.1 µg/m^3^ during the 7 days before delivery, and 25.6 ± 8.6 µg/m^3^ for the last month of pregnancy (Table 3). Average trimester-specific PM_10_ exposure was 21.5 ± 5.1 µg/m^3^ for the first trimester, 22.3 ± 4.3 µg/m^3^ for the second trimester, and 24.4 ± 5.7 µg/m^3^ for the third trimester. The average distance from the participant’s home address to the nearest major road was 207 m (IQR = 85–676 m).

**Table 3:** Exposure characteristics.

Pollution indicator	Meana ± SD		25th percentile	75th percentile
PM10 (µg/m³)							
Week (mean lag 0–7)		24.9 ± 11.1			17.5		30.5
Last month		25.6 ± 8.6			19.3		29.5
Trimester 1		21.5 ± 5.1			18.1		23.7
Trimester 2		22.3 ± 4.3			19.3		25.3
Trimester 3		24.4 ± 5.7			20.2		28.1
Whole pregnancy		22.7 ± 3.7			20.1		25.1
Traffic-related pollution							
Residential distance to major road (m)		207			85		676
aArithmetic mean except for the residential distance to major road, for which the geometric mean is given.							

*Predictors of mtDNA content.* Placental mtDNA content was negatively associated with parity (β = –0.064 ± 0.027, *p* = 0.018), also after adjusting for maternal age (β = –0.063 ± 0.029, *p* = 0.028), negatively associated with cold season (β = –0.243 ± 0.040, *p* < 0.0001), and positively associated with apparent temperature the week before delivery (β = 0.018 ± 0.003, *p* < 0.0001). Birth weight adjusted for newborn’s sex, gestational age, season, and apparent temperature during the third trimester was not significantly associated with placental mtDNA content (*p* = 0.71) nor with PM_10_ exposure during the third trimester (*p* = 0.33).

**Figure 1:**
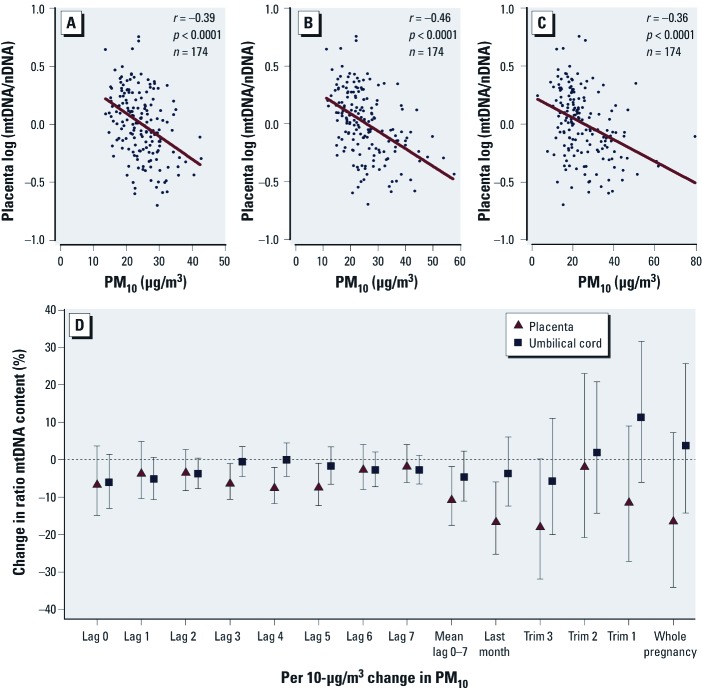
Association between mtDNA content in placental tissue or cord blood and PM_10_ exposure. Three correlation plots indicate PM_10_ exposure during a period of pregnancy: third trimester (*A*), the last month of pregnancy (*B*), and mean of 7 days before delivery (*C*). (*D*) The percent change (95% CI) in mtDNA content of both placental tissue and cord blood for each 10-µg/m^3^ increase of PM_10_ exposure. The model is adjusted for newborn’s sex, maternal age, parity, gestational age, ethnicity, smoking status, season, and time-specific apparent temperature. Additionally, umbilical cord blood was adjusted for blood cell count (number of white blood cells, percent neutrophils, and number of platelets). Values of mtDNA content are log transformed.

*mtDNA content in association with* in utero *PM_10_ exposure.* Unadjusted analysis showed that placental mtDNA content was correlated with PM_10_ exposure during the whole pregnancy (β = –0.01 ± 0.006, *p* = 0.068), but this was largely attributable to exposure during the last week, last month, and third trimester of pregnancy (all *p* < 0.0001, Figure 1A–C). PM_10_ exposures during the first and second trimester of pregnancy were not significantly (*p* > 0.31) associated with placental mtDNA content. We adjusted for relevant variables that may influence the mtDNA content outcome (*p* ≤ 0.10). Although newborn’s sex, maternal age, smoking status, gestational age, and ethnicity were not significantly associated with placental mtDNA content, we forced these variables into the regression model, together with parity, season, and time-specific apparent temperature. After adjustment for the aforementioned variables, placental mtDNA content remained negatively associated with PM_10_ exposure during the last week, last month, and third trimester of pregnancy (Figure 1D). Each 10-µg/m^3^ increase in PM_10_ was associated with a lower placental mtDNA content of 10.1% [95% confidence interval (CI): –17.6, –1.9, *p* = 0.02] when considering the average exposure during the last week of pregnancy, 16.1% (95% CI: –25.2, –6.0, *p* = 0.003) during the last month of pregnancy and 17.4% (95% CI: –31.8, –0.1, *p* = 0.05) during the third trimester (Table 4). Placental mtDNA content at birth did not correlate with PM_10_ exposure during first and second trimester.

**Table 4:** Estimated change in placental and umbilical cord blood mtDNA content in association with PM_10_ during pregnancy or distance from residence to nearest major road.

Placental tissue (n = 174)					Umbilical cord blood (n = 176)				
Variable	Percent change	95% CI	p-Value	Percent change	95% CI	p-Value
Time window (PM10)a,b												
Trimester 1		–10.9		–27.1, 8.9		0.26		11.2		–6.0, 31.6		0.22
Trimester 2		–1.3		–20.7, 22.9		0.91		1.8		–14.2, 20.8		0.84
Trimester 3		–17.4		–31.8, –0.1		0.05		–5.8		–20.0, 11.0		0.48
Last month		–16.1		–25.2, –6.0		0.003		–3.6		–12.3, 6.0		0.45
Week (mean lag 0–7)		–10.1		–17.6, –1.9		0.02		–4.6		–11.0, 2.2		0.20
Traffic												
Distance from residence to a major roadb,c		4.0		0.4, 7.8		0.03		–2.0		–4.8, 0.9		0.17
aEffect size was estimated for each 10-µg/m3 increase in PM10 exposure at mother’s residence during the corresponding period. bAdjusted for newborn’s sex, maternal age (years), parity (continuous), gestational age (weeks), ethnicity (European/non-European), smoking status (never, before, current), season (cold/warm period), and time-specific apparent temperature (°C). Additionally, umbilical cord blood was adjusted for blood cell count (number of white blood cells, percent neutrophils, and number of platelets). cPercentage was calculated for each doubling in distance from residence to major road (based on a model with log distance and log mtDNA content).												

In contrast to placental mtDNA content, none of these pollution windows were significantly associated with cord blood mtDNA content, either before or after adjusting for potential confounders as in the previous models and also including platelet counts, neutrophils, and total number of white blood cells (Table 4, Figure 1D). Although we adjusted for cord blood cell distribution, mtDNA content was not significantly associated with cord blood platelets (*p* = 0.97), neutrophils (*p* = 0.47), white blood cells (*p* = 0.18), or the white blood cell/platelet ratio (*p* = 0.15).

*Markers of traffic-related air pollution.* Distance to a major road is an exposure marker that can be used as a surrogate for traffic-related air pollution (Hoek et al. 2002). Adjusted estimates showed that distance to major roads was significantly associated with placental mtDNA content—a 4% increase in mtDNA content with each doubling of the distance (95% CI: 0.4, 7.8) (Table 4). No association was observed between cord blood mtDNA content and distance to major roads.

*Sensitivity analysis.* Excluding women with preeclampsia (*n =* 1) or other pregnancy complications (*n =* 2) did not alter the reported changes between PM_10_ exposure and mtDNA content of placental tissue or cord blood. The expression of the nuclear genes, used as internal controls to quantify mtDNA content in qPCR assays, was not significantly associated with PM_10_ exposure during the various time windows (*p* > 0.28). Models in which we replaced PM_10_ exposure with NO_2_ exposure [see Supplemental Material, Tables 1 and 2 (http://dx.doi.org/10.1289/ehp.1104458)] showed significant negative associations with placental mtDNA content for a 10-µg/m^3^ increase in NO_2_ during the last month (–14.1%; 95% CI: –26.5, –0.3, *p* = 0.05) and third trimester (–21.8%; 95% CI: –32.1, –9.8, *p* = 0.0009) of pregnancy. The other time periods were not significantly associated, and no significant associations between NO_2_ and cord blood mtDNA content during any time period were observed.

## Discussion

The placenta plays a pivotal role in nutrient transfer, growth, and organ development and these processes are regulated by mitochondria. Placental mitochondria also play an important role in the proper formation and functioning of the placenta, and therefore are essential for fetal health. Urban PM has adverse effects on the functional morphology of the placenta in experimental animal models (Veras et al. 2008) and has been associated with adverse health outcomes of the fetus (Ballester et al. 2010; Dejmek et al. 1999; Gemma et al. 2006; Glinianaia et al. 2004; Kannan et al. 2006; Morello-Frosch et al. 2010), but the molecular changes have barely been studied. The key finding of our study is that placental mtDNA content, a molecular marker of mitochondrial damage and mitochondrial inflammation, is associated with *in utero* exposure to PM_10_, especially during the last period of pregnancy. We also assessed the association between proximity of the mother’s home to major roads, as a surrogate of traffic-related air pollution and the placental mtDNA content. The placental mtDNA content was positively associated with an increase in residential distance to major roads. These associations persisted with adjustment for newborn’s sex, maternal age, smoking status, gestational age, ethnicity, parity, season, and time-specific apparent temperature or any other covariate studied. In contrast to placental mtDNA content, none of these average pollution levels correlated with mtDNA content from cord blood.

Our observation that exposure to PM_10_ and traffic-related air pollutants during pregnancy appears to modulate mtDNA replication in a negative manner is consistent with two studies on the effects of smoking. A decrease in the mtDNA content in the lungs of smokers has been observed, which was attributed to the oxidative stress induced by smoking (Lee et al. 1998). Moreover, Bouhours-Nouet et al. (2005) showed that maternal smoking is associated with mtDNA depletion of placental tissue from newborns. In our study, we did not confirm a significant association between smoking during pregnancy and a lower mtDNA content (*p* = 0.40) in the whole study population, possibly due to the low prevalence of maternal smoking (15.7%).

The biological mechanisms by which air pollution may affect fetal health outcomes are poorly understood, but the formation of ROS and inflammation due to PM is thought to be of importance. In addition to ROS formed as a by-product of mitochondrial respiration (Li et al. 2003), ROS may also be present in mitochondria of placental tissue in response to maternal smoking (Bouhours-Nouet et al. 2005). However, ROS are formed not only in placental mitochondria but also in mitochondria of endothelial cells, lining the inside of maternal and fetal capillary surface areas of the placenta. It has been shown that PM exposure, particularly to pro-oxidative combustion particles, influences endothelial function (Li et al. 2006; Peretz et al. 2008). The observation that smoke exposure during pregnancy causes a direct increase in the vascular resistance of the placenta from the fetal side (Geelhoed et al. 2011; Larsen et al. 2002) suggests that PM exposure may lead to an increased resistance of umbilical–placental circulation that may impair oxygen and nutrient exchange across the placenta. Mitochondria respond to energy deficiency by synthesizing more copies of their mtDNA and increase their abundance (Hou et al. 2010). However, mtDNA is particularly vulnerable to ROS-induced damage and has a high mutation rate (Linnane et al. 1989). mtDNA replication can be a compensatory mechanism in response to inefficient mitochondrial function due to mutations, resulting in a vicious circle of more ROS formation from defective cells (Andreu et al. 2009). In time, the bioenergetic and replicative functions of defective mitochondria decline, resulting in further depletion of mtDNA content and loss of mitochondrial function (Wong et al. 2009).

Pollutants may interfere differently with placental development during different gestational periods. During the first trimester and late pregnancy, the placenta expresses several cytochrome P450 enzymes, although only a few of them are active, indicating that metabolism of PM may be reduced (Myllynen et al. 2005). Mutations in placental mtDNA may occur in early pregnancy, leading to an onset of mitochondrial dysfunction in later trimesters. The strongest association we observed between placental mtDNA content and different PM_10_ exposure windows during pregnancy was for the last period of pregnancy, suggesting that this might be a potential window for susceptibility to PM_10_ exposure. Indeed, first- and third-trimester air pollution exposures have been implicated as having the most relevance for low birth weight and preterm birth (Gehring et al. 2011). The study by Morello-Frosch et al. (2010) revealed a decrease in birth weight of 7.7 g for each 10-µg/m^3^ increase in PM_10_ in the third trimester, although the international collaboration on air pollution and pregnancy outcomes reported heterogeneity in estimated effects of air pollution on birth weight among different locations (Parker et al. 2011). We found neither a significant association between birth weight and PM_10_ exposure nor an association between birth weight and placental and cord blood mtDNA content.

Mitochondrial dysfunction can be caused by a change in mtDNA content and may be related to the development of multiple forms of disease. Decreased mtDNA content of white blood cells has been shown in type 2 diabetes (Choi et al. 2001; Gianotti et al. 2008; Wong et al. 2009), breast cancer (Xia et al. 2009; Yu et al. 2007), and low birth weight (Gemma et al. 2006). Alternatively, low-dose benzene exposure in various occupational groups and PM exposure in steelworkers was associated with damaged mitochondria, as exemplified by increased mtDNA copy numbers in whole blood and white blood cells, respectively (Carugno et al. 2012; Hou et al. 2010). In contrast to these observations, our results are consistent with those of an earlier report (Bouhours-Nouet et al. 2005) on maternal smoking (a personalized form of air pollution) and a lower mtDNA content. We must bear in mind that mtDNA content fluctuates during aging, under the influence of different environmental factors and the tissue investigated (Andreu et al. 2009; Clay Montier et al. 2009). Experimental evidence shows that short telomeres trigger a decline in mitochondrial mass that induces additional telomere shortening (Sahin et al. 2011).

The fact that we observed associations with mtDNA content in placental tissue but not in umbilical cord blood demands consideration. First, umbilical cord blood has a separate circulation that may not be representative of other tissues. For example, Gemma et al. (2006) postulated that umbilical cord blood is not representative for fetal tissue. Also, they found no association between maternal leukocyte mtDNA content and umbilical cord mtDNA content, indicating that leukocyte mtDNA of cord blood may not be a good indicator of mtDNA in maternal tissue or that other *in utero* factors influence mtDNA content of cord blood. Some authors attributed variation in mtDNA in human blood cells to variation in platelets (Banas et al. 2004; Cossarizza et al. 2003). Platelet contamination increases mtDNA without contributing to nDNA and affects the mtDNA content. However, we adjusted our models of cord blood mtDNA content for blood cell count (including platelet count). A second consideration is that the movement of pollutants into the fetal compartments can be blocked or facilitated by placental transporters (Adibi et al. 2010; Veras et al. 2008). Pollutants that do not traverse the placenta will not affect the cord blood but may affect placental cells, including active nutrient transfer and vascular development that may adversely affect fetal development. Complex vascular alterations are considered to be the main cause of placental abnormalities in the second and third trimester, and PM-induced effects may be a mechanism by which these alterations occur (Jalaludin et al. 2007). Finally, differences in turnover rates of mtDNA between tissues have been documented (Collins et al. 2003; Gross et al. 1969) which might also contribute to different effects on cord blood and placental tissue.

Morphological changes in placental structure and vasculature occur throughout the whole pregnancy (Myllynen et al. 2005). Peroxisome proliferator activated receptor protein gamma (PPARγ), a nuclear transcription factor, and its transcriptional coactivator PPARγ-coactivator alpha (PGC-1α) are essential in basic placental development and function through the regulation of genes involved in trophoblast differentiation, angiogenesis, fatty acid transport, and inflammation (Adibi et al. 2009). In addition, PGC-1α also controls other nuclear receptors and transcription factors that are essential in mitochondrial biogenesis and energy metabolism (Wenz 2011). We postulate that PM-induced oxidative stress may have effects on the expression of PPARγ and PGC-1α, resulting in changes in expression of PPARγ-dependent genes and genes controlling mitochondrial biogenesis and mtDNA content of placental mitochondria.

Several limitations of the present study warrant consideration. Although our results were consistent after multiple adjustments, we cannot exclude the possibility of residual confounding by some unknown factor that is associated with both mitochondrial function and ambient air pollution. Ambient exposure does not account for indoor exposure, but we obtained information on environmental tobacco smoke. mtDNA content showed considerable variation within placenta (19.3%); therefore we took biopsies at a fixed location to minimize variation of placental mtDNA content attributed to differences across placental regions.

## Conclusion

In conclusion, *in utero* PM_10_ exposure during the last period of pregnancy was associated with mitochondrial damage as exemplified by placental mtDNA content. This might suggest a potential window for susceptibility to PM_10_ exposure. The potential health consequences of decreased mtDNA content in early life must be further elucidated.

## Supplemental Material

(147 KB) PDF
