# Supplementary material for: Placental Mitochondrial DNA Content and Particulate Air Pollution during in Utero Life
Source: Environ Health Perspect. 2012 May 24;120(9):1346–52. doi: 10.1289/ehp.1104458 (PMC3440109; doi:10.1289/ehp.1104458)
Supplement: (147 KB) PDF [file ehp.1104458.s001.pdf]

Supplemental Materials:

Placental Mitochondrial DNA Content and Particulate Air Pollution During *in Utero* Life

Bram G. Janssen[1], Elke Munters[1], Nicky Pieters[1], Karen Smeets[1], Bianca Cox[1], Ann Cuypers[1], Frans Fierens[2], Joris Penders[3], Jaco Vangronsveld[1], Wilfried Gyselaers[4], Tim S. Nawrot[1,5]

[1] Centre for Environmental Sciences, Hasselt University, Diepenbeek, Belgium

[2] Belgian interregional Environment Agency, Brussels, Belgium

[3] Biomedical Research Institute, Hasselt University, Diepenbeek, Belgium

[4] Department of Obstetrics, East-Limburg Hospital, Genk, Belgium

[5] Department of Public Health, Occupational and Environmental Medicine, Leuven University (KULeuven), Leuven, Belgium

Table of Contents:

Supplemental Materials, Table 1 .... pg. 2

Supplemental Materials, Table 2 .... pg. 3

Supplemental Materials, Table 1. Exposure characteristics of nitrogen dioxide.

Pollution indicator	Mean[a]	SD	25th percentile	75th percentile
Nitrogen dioxide (NO_2), µg/m³				
Week (Mean Lag 0-7)	22.0	7.8	16.5	26.7
Last month	22.9	7.5	17.5	27.8
Trimester 1	20.1	5.9	16.2	23.6
Trimester 2	21.6	5.9	17.4	25.5
Trimester 3	22.9	7.0	18.4	27.2
Whole pregnancy	21.5	5.0	18.6	24.5

[a] NO_2 values are presented as arithmetic mean with standard error and 25th-75th percentile.

Supplemental Materials, Table 2. Estimated change in placental and umbilical cord blood mtDNA content in association with NO_2 during pregnancy or distance from residence to nearest major road.

	Placental tissue (*n* = 174)			Umbilical cord blood (*n* = 176)		
Variable	Percentage change	95% CI	*p*-value	Percentage change	95% CI	*p*-value
Time window, NO_2 [a, b]						
Trimester 1	14.1%	-2.5 to 33.4%	0.10	6.0%	-6.1 to 20.0%	0.38
Trimester 2	-9.6%	-23.0 to 6.1%	0.22	6.8%	-6.0 to 20.9%	0.30
Trimester 3	-21.8%	-32.1 to -9.8%	0.0009	4.4%	-6.6 to 16.7%	0.44
Last month	-14.4%	-26.5 to -0.3%	0.05	6.2%	-5.5 to 19.3%	0.29
Week (Mean Lag 0-7)	-3.0%	-16.0 to 12.0%	0.68	-2.3%	-12.4 to 9.0%	0.68

[a] Effect size was estimated for each 10 µg/m^3 increase in NO_2 exposure at mother's residence during the corresponding period.

[b] Adjusted for newborn's sex (boy/girl), maternal age (years), parity (continuous), gestational age (weeks), ethnicity (European/non-European), smoking status (never, before, current), season (cold/warm period) and apparent week temperature (°C). Additionally, umbilical cord blood was adjusted for blood cell count (# of white blood cells, % neutrophils and # of platelets).
